# Retroperitoneal biloma—A rare differential diagnosis of perirenal fluid accumulation

**DOI:** 10.1002/iju5.12723

**Published:** 2024-03-23

**Authors:** Kazushi Hanawa, Masanari Fukasawa, Tadashi Aoki, Munehiro Nozawa, Yoshio Takihana, Yuji Mishina, Hiroshi Nakagomi

**Affiliations:** ^1^ Department of Urology Yamanashi Kosei Hospital Yamanashi City Yamanashi Japan; ^2^ Department of Gastroenterology Yamanashi Kosei Hospital Yamanashi City Yamanashi Japan

**Keywords:** hepatic duct, perinephric abscess, retroperitoneal biloma

## Abstract

**Introduction:**

Nontraumatic biliary rupture and retroperitoneal biloma infrequently occur. Here, we report a case of retroperitoneal biloma due to spontaneous left hepatic duct perforation, which was difficult to differentiate from a perirenal abscess.

**Case presentation:**

A 94‐year‐old female patient was hospitalized with symptoms of fatigue and right back pain that lasted for 5 days. Computed tomography revealed fluid accumulation in the retroperitoneum, and urinary extravasation and right perinephric abscess were suspected. Antimicrobial treatment and drainage with ureteral stents and urethral catheters demonstrated no symptom improvement. Ultrasound‐guided puncture of the abscess revealed the presence of bile. Pigtail catheter drainage improved symptoms and inflammatory response. After diagnosis, endoscopic retrograde cholangiopancreatography revealed bile leakage, and a bile duct stent was inserted.

**Conclusion:**

Biloma can cause perirenal fluid accumulation, and they should be considered an origin of perirenal fluid accumulation when urinary tract lesions are excluded.

**Abbreviations & Acronyms**
CT: computed tomography
EGPD: echo‐guided percutaneous drainage
ERCP: endoscopic retrograde cholangiopancreatography
HU: hounsfield unit

**Keynote message**

Retroperitoneal biloma requires differentiation from urological disease.

## Introduction

Biloma develops because of bile accumulation outside the biliary ducts. Most causes are medical or traumatic, such as surgical or endoscopic, and spontaneous perforation rarely occurs.^1^ Biloma formation is caused by a lesion in the biliary tree, which can include intrahepatic and extrahepatic lesions. Biloma occurs intrahepatic, intraperitoneal, or retroperitoneal, and most rarely within the retroperitoneum.^1^ To date, the literature has published 10 cases, reporting retroperitoneal biloma due to spontaneous bile duct perforation,^2^, ^3^, ^4^, ^5^, ^6^, ^7^, ^8^, ^9^, ^10^, ^11^ with only one case due to left hepatic duct perforation.^11^ Here, we report a case of retroperitoneal biloma due to left hepatic duct perforation, which required differentiation from perirenal abscess and urinary extravasation.

## Case presentation

**Fig. 1:**
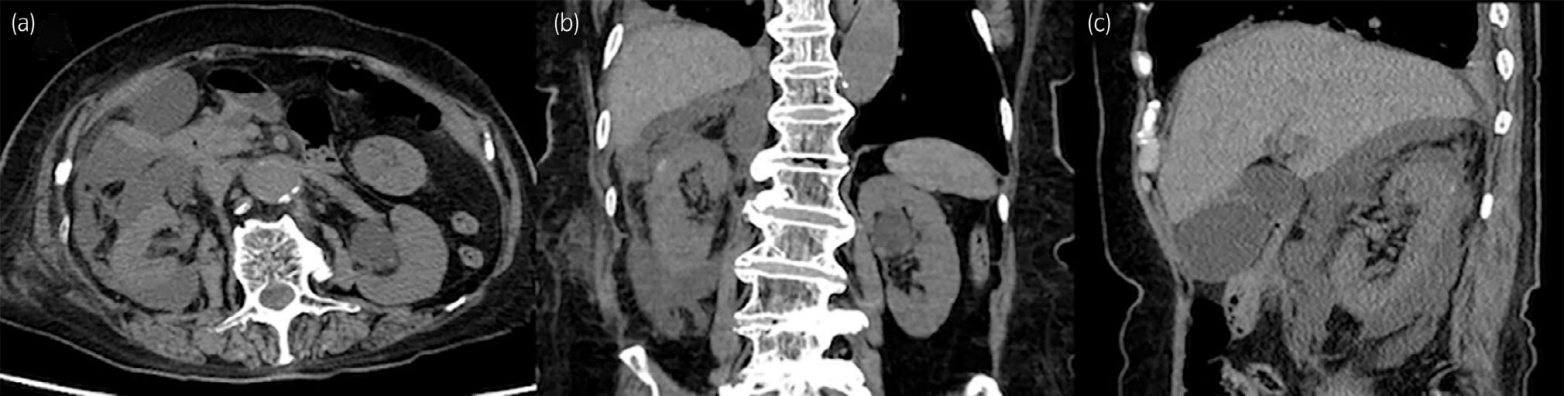
CT revealed fluid accumulation in the retroperitoneum and an increased concentration of right perirenal fatty tissue. (a) Axial section, (b) coronal section, (c) sagittal section.

**Fig. 2:**
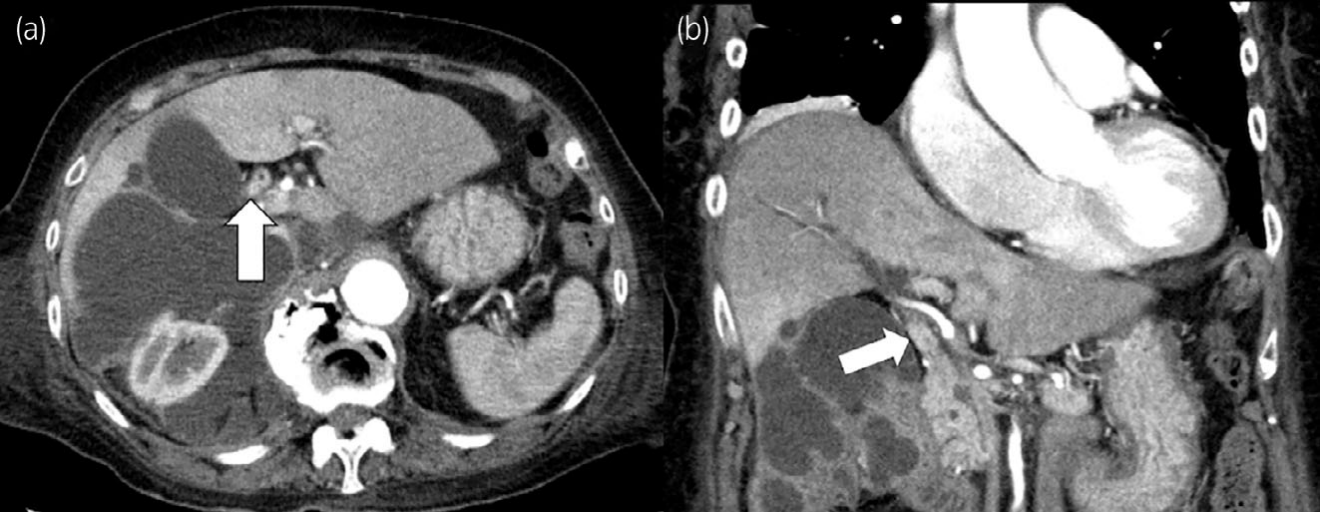
Contrast‐enhanced CT of the abdomen showed an increase in accumulation of multifocal fluid with septal contrast effect. Soft tissue in the common bile duct demonstrated contrast effects (arrows). (a) Axial section, (b) coronal section.

**Fig. 3:**
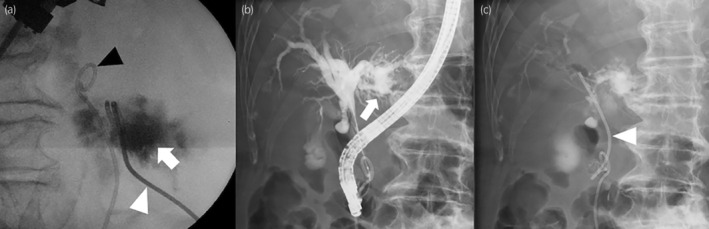
(a) Contrast spread within the retroperitoneal biloma. A pigtail catheter was inserted into the perirenal fluid for continuous drainage (arrow, biliary discharge; black arrowhead, ureteral stent; white arrowhead, pigtail catheter). (b) Cholangiography revealed a contrast leakage (arrow) from hepatic duct of the left lobe. (c) A stent (arrowhead) was inserted into the common bile duct.

A 94‐year‐old female patient presented to the emergency room with constant fever, fatigue, and right back pain. Vital signs upon visit included a temperature of 36.0°C, heart rate of 57 bpm, blood pressure of 100/62 mmHg, and SpO_2_ of 99% (room air). Her medical history included hypertension, osteoporosis, atrial fibrillation, and cataracts. The patient reported no history of abdominal or thoracic surgery or trauma. Laboratory workup revealed significantly elevated bilirubin level, cholestasis, and increased inflammation markers (Table 1). Urinalysis revealed pyuria and bacteriuria. Abdominal ultrasound detected a cystic mass with a septum surrounding the right kidney. Abdominal plain CT revealed fluid accumulation in the retroperitoneum and elevated right perirenal fatty tissue concentration, which indicated a right perirenal abscess (Fig. 1). The patient was hospitalized for antimicrobial treatment. Abdominal plain CT after 6 days revealed increased perirenal fluid accumulation and right pyelectasia. The patient consulted with us regarding urinary extravasation. The excretory phase of contrast‐enhanced CT was not evaluated prior to the operation. Although ureteral stenting was performed for urinary extravasation treatment, retrograde pyelography did not reveal contrast leakage at that time. Right back pain continued on the third postoperative day. Abdominal contrast‐enhanced CT revealed an abscessification because of an increase in multifocal fluid accumulation with septal contrast effect, and soft tissue in the common bile duct demonstrated contrast effects, which indicated a common bile duct tumor (Fig. 2). Blood tests revealed an increase in the levels of biliary tract enzymes and inflammatory markers (Table 1). EGPD was performed on day 11 of admission. Puncture drainage demonstrated biliary discharge and total bilirubin of 4.96 mg/dL and creatinine of 0.72 mg/dL in biochemical tests, which caused the retroperitoneal biliary leakage diagnosis. A pigtail catheter was inserted for continuous drainage (Fig. 3a), which improved symptoms. On day 16 of admission, blood tests revealed improvement in bilirubin level, cholestasis, and inflammation markers (Table 1). ERCP was performed on the same day, and cholangiography revealed a contrast leakage from the hepatic duct of the left lobe, causing a diagnosis of retroperitoneal biloma due to left hepatic duct rupture (B2 or B3) caused by a common bile duct tumor (Fig. 3b). A bile duct stent was inserted, and the patient demonstrated a good course (Fig. 3c).

**Table 1:** Laboratory data at admission and prior to EGPD and ERCP

Parameter	On admission	Before EGPD	Before ERCP	Reference range
Total bilirubin	3.15	2.21	0.87	0.40–1.50 mg/dL
Alkaline phosphatase level	147	333	189	38–113 U/L
Gamma‐glutamyl transferase	117	262	152	13–64 U/L
Aspartate aminotransferase	30	51	43	13–30 U/L
Alanine aminotransferase	55	32	30	10–42 U/L
Lactate dehydrogenase	369	206	191	124–222 U/L
Blood urea nitrogen	76.6	20.6	12.4	8.0–20.0 mg/dL
Creatinine	2.28	0.85	0.65	0.65–1.07 mg/dL
C‐reactive protein	27.99	13.9	4.18	≦0.14 mg/dL
White blood cell	9,500	16,000	6,230	3300–8600/μL
Procalcitonin	4.75	0.33	0.19	<0.50/mL

## Discussion

Biloma is the collection of bile outside the biliary tree and can be either encapsulated or not. The formation is the result of a lesion in the biliary tree, which can be either intrahepatic or extrahepatic.^1^ Retroperitoneal biloma is a fluid accumulation that surrounds the kidney. The differentiation of retroperitoneal biloma includes hematoma, retroperitoneal abscess due to liver abscess rupture, lymphangioma, perinephric abscess, and urinary extravasation. The following several causes of spontaneous bile duct perforation have been proposed: (i) erosion by biliary stones that injured the duct wall; (ii) increased intraductal pressure due to a distal bile duct obstruction (by stones, or carcinomas), and others.^11^ Generally, urinary extravasation is differentiated in the excretion phase of contrast‐enhanced CT, but not perirenal abscesses and retroperitoneal biloma because of similar results. Previous literature has revealed a lower CT value of bile than that of abscess.^12^ CT values may help differentiate the abscess from bile and urine. CT values of bile, urine, and perirenal effusion in this case (9.7 HU, 9.2 HU, and 11.1 HU) indicated that the perirenal fluid accumulation was not an abscess. Conversely, differentiating them by CT alone is difficult because of the complex anatomy of the retroperitoneum. Magnetic resonance imaging shows that bile, urine, and abscess are all high‐intensity signals on T2‐weighted intensity and low‐intensity signals on T1‐weighted intensity, which are not useful for differentiation. Magnetic resonance cholangiopancreatography evaluates bile duct stricture, and hepatobiliary iminodiacetic acid scan is a useful and recommended imaging test for bile leakage identification.^13^ Retroperitoneal biloma demonstrated atypical clinical symptoms, and it is frequently difficult to make a definitive diagnosis based on imaging only. However, biloma should be considered in cases with total bilirubin elevation because, notably, the total bilirubin is above the baseline value in all cases in the previous literature.^7^, ^8^, ^9^, ^10^, ^11^

The previous literature observed several cases in which the difficulty of diagnosis resulted in open surgery and intraoperative diagnosis.^5^, ^7^, ^9^, ^11^ Open surgery is invasive and should be avoided if possible. Percutaneous drainage is a minimally invasive and reliable diagnostic and therapeutic procedure. Further, previous literature supports aggressive percutaneous drainage because of the large number of cases with a course that requires percutaneous drainage as treatment^3^, ^5^, ^8^, ^10^, ^11^ and the necessity for rapid diagnosis because of the life‐threatening disease when it becomes severe. We acknowledge a report of good outcomes with a conservative approach using ERCP and bile duct stents after percutaneous drainage diagnosis.^10^ Conversely, ERCP and stenting should generally only be used as temporary measures to manage the acute obstructive phase^13^ and surgical intervention is necessary after biliary decompression if the findings do not improve. The hemodynamic status of this patient was stable compared with previous reports. A good course was achieved with puncture drainage and bile duct stent in this case. We consider appropriate management to be required for the condition of each case.

## Conclusion

Biloma can cause perirenal fluid accumulation, and they should be considered an origin of perirenal fluid accumulation when urinary tract lesions are excluded.

## Author contributions

Kazushi Hanawa: Validation; visualization; writing – original draft; writing – review and editing. Masanari Fukasawa: Writing – review and editing. Tadashi Aoki: Writing – review and editing. Munehiro Nozawa: Writing – review and editing. Yoshio Takihana: Writing – review and editing. Yuji Mishina: Writing – review and editing. Hiroshi Nakagomi: Project administration; supervision; writing – review and editing.

## Conflict of interest

The authors declare no conflict of interest.

## Approval of the research protocol by an Institutional Reviewer Board

Not applicable.

## Informed consent

Not applicable.

## Registry and the Registration No. of the study/trial

Not applicable.

## Compliance with ethical standards

All procedures performed in this study involving human participants were in accordance with the ethical standards of the institutional and/or national research committee and with the 1964 Helsinki Declaration and its later amendments or comparable ethical standards.
